# Author Correction: Transcriptional synergy between Tat and PCAF is dependent on the binding of acetylated Tat to the PCAF bromodomain

**DOI:** 10.1038/s44318-025-00612-z

**Published:** 2025-12-16

**Authors:** Alexander Dorr, Veronique Kiermer, Angelika Pedal, Hans-Richard Rackwitz, Peter Henklein, Ulrich Schubert, Ming-Ming Zhou, Eric Verdin, Melanie Ott

**Affiliations:** 1 Deutsches Krebsforschungszentrum (DKFZ), Heidelberg, 69120 Germany; 2 Gladstone Institute of Virology and Immunology, University of California, San Francisco, CA 94141 USA; 3 Humboldt University, Institute of Biochemistry, D-10115 Berlin, Germany; 4 Laboratory of Viral Diseases, National Institutes of Health, Bethesda, MD 20892 USA; 5 Structural Biology Program, Department of Physiology and Biophysics, Mount Sinai School of Medicine, New York, NY 10029-6574 USA

## Abstract

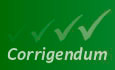

**Correction to:** *The EMBO Journal* (2002) 21:2715–2723. 10.1093/emboj/21.11.2715 | Published online 3 June 2002

The authors contacted the journal after being made aware of blot re-use within Fig. 5A. The authors were able to locate the original scans and the correct blots. After reviewing the data provided by the authors, the journal retracts and replaces the following figure.

**Figure 5A is retracted and replaced**.

**Figure 5A:**
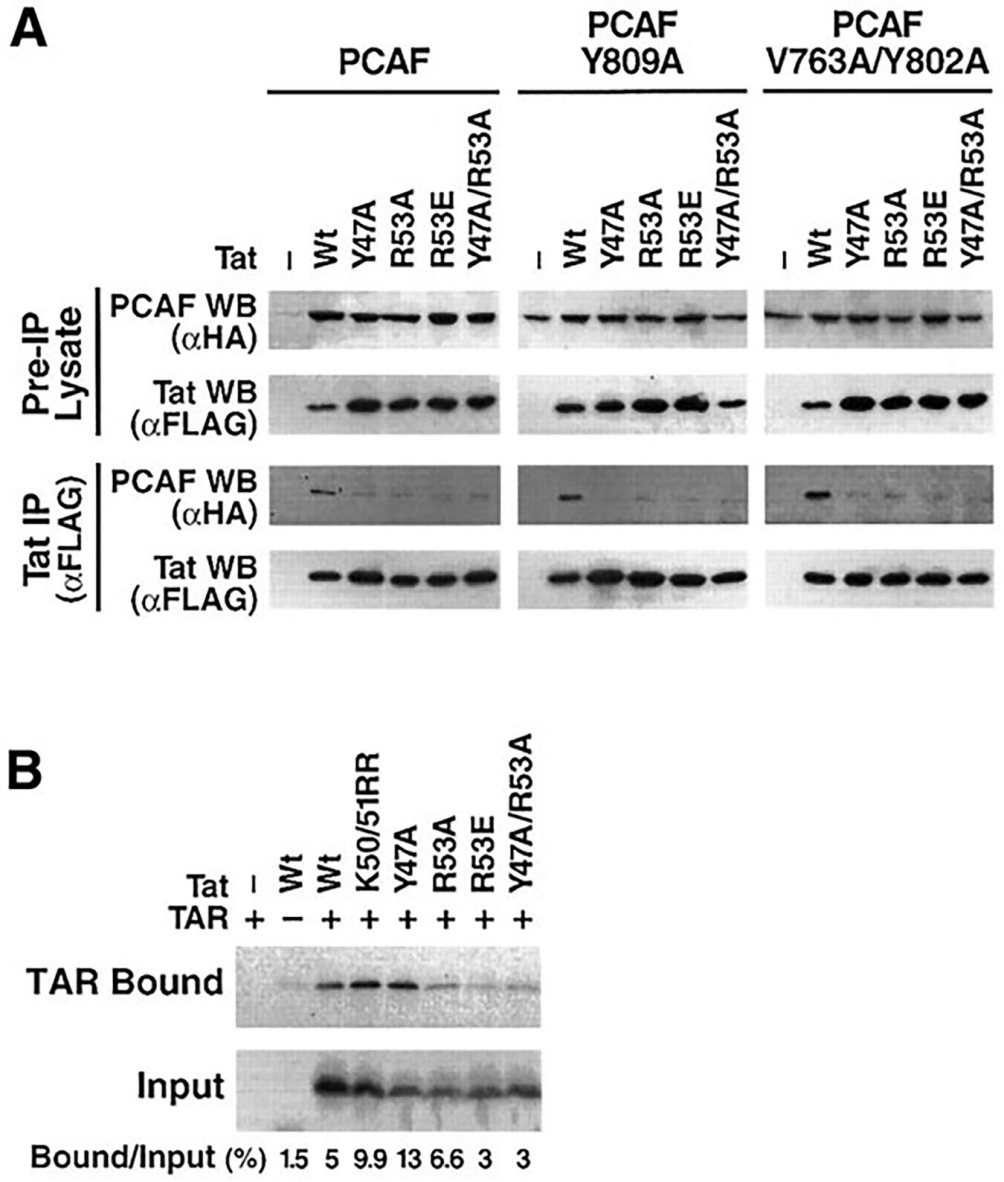
Original.

**Figure 5A:**
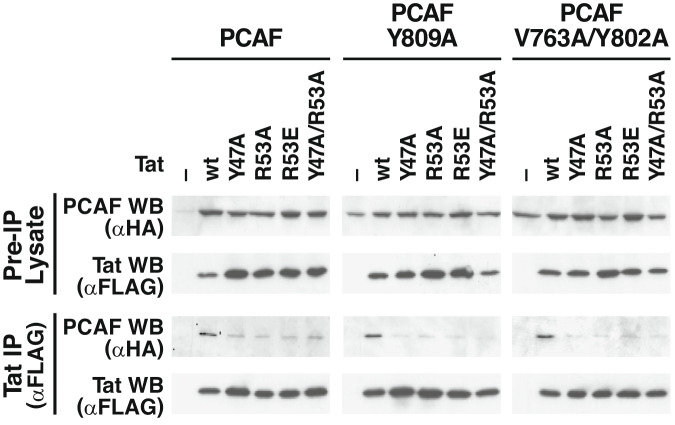
**Corrected.** Source data are available online for this figure.

**The original compositor data sheet for Fig. 5A is published with this correction**.

**Associated original scans are published with this correction**.

Author statement.

During assembly of Fig. 5, the researcher provided a guide file, ‘01.0324E.Grant.pdf,’ which contained gel section IDs corresponding to the file names of the linked images. The compositor inadvertently copied the incorrect gel for Fig. 5, 2nd row, 3rd column, using ‘01-0324D (01-0323P GelSec)’ instead of the correct ‘01-0324D (01-0323Q GelSec).’

The corrigendum does not affect the findings and conclusions of the manuscript.

AD, CK, US, M-MZ, EV and MO agree to this correction. No response could be obtained from AP, H-RR & PH.

## Supplementary information

01.0324E.Grant

SOURCE DATA 01.0323 E_Verdin

