# Supplementary material for: Author Correction: Transcriptional synergy between Tat and PCAF is dependent on the binding of acetylated Tat to the PCAF bromodomain
Source: EMBO J. 2025 Dec 16;45(2):633–5. doi: 10.1038/s44318-025-00612-z (PMC12811238; doi:10.1038/s44318-025-00612-z)
Supplement: Supplementary file 1 — 01.0324E.Grant [file 44318_2025_612_MOESM1_ESM.pdf]

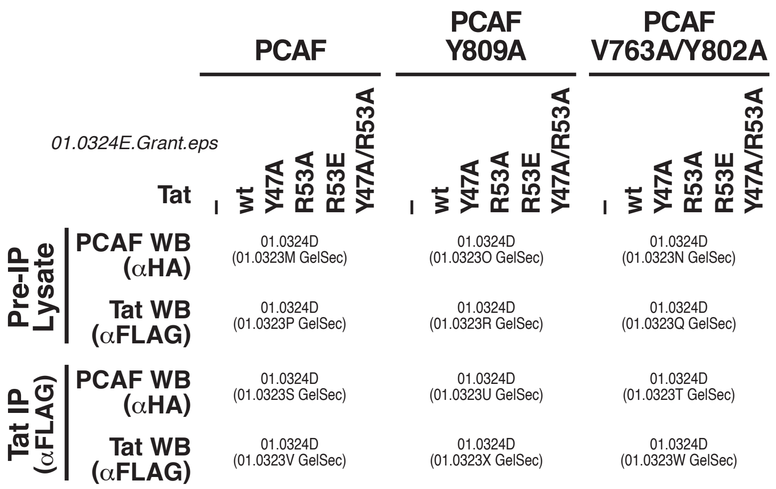
PCAF
PCAF Y809A
PCAF V763A/Y802A
01.0324E.Grant.eps
Tat
–
wt
Y47A
R53A
R53E
Y47A/R53A
Pre-IP Lysate
PCAF WB (αHA)
01.0324D (01.0323M GelSec)
01.0324D (01.0323O GelSec)
01.0324D (01.0323N GelSec)
Tat WB (αFLAG)
01.0324D (01.0323P GelSec)
01.0324D (01.0323R GelSec)
01.0324D (01.0323Q GelSec)
Tat IP (αFLAG)
PCAF WB (αHA)
01.0324D (01.0323S GelSec)
01.0324D (01.0323U GelSec)
01.0324D (01.0323T GelSec)
Tat WB (αFLAG)
01.0324D (01.0323V GelSec)
01.0324D (01.0323X GelSec)
01.0324D (01.0323W GelSec)
