# Supplementary figures and images for: Author Correction: Transcriptional synergy between Tat and PCAF is dependent on the binding of acetylated Tat to the PCAF bromodomain
Source: EMBO J. 2025 Dec 16;45(2):633–5. doi: 10.1038/s44318-025-00612-z (PMC12811238; doi:10.1038/s44318-025-00612-z)

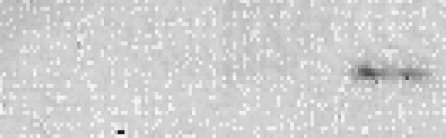

Supplement: Supplementary file 2 — SOURCE DATA 01.0323 E_Verdin [file 44318_2025_612_MOESM2_ESM.zip › SOURCE DATA 01.0323 E_Verdin/01.0323 H E_Verdin.tif]

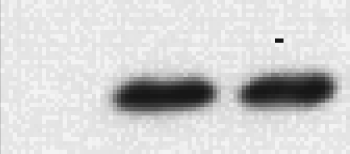

Supplement: Supplementary file 2 — SOURCE DATA 01.0323 E_Verdin [file 44318_2025_612_MOESM2_ESM.zip › SOURCE DATA 01.0323 E_Verdin/01.0323 L E_Verdin.tif]

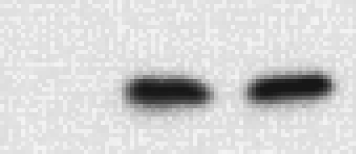

Supplement: Supplementary file 2 — SOURCE DATA 01.0323 E_Verdin [file 44318_2025_612_MOESM2_ESM.zip › SOURCE DATA 01.0323 E_Verdin/01.0323 K E_Verdin.tif]

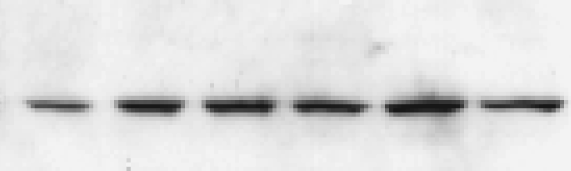

Supplement: Supplementary file 2 — SOURCE DATA 01.0323 E_Verdin [file 44318_2025_612_MOESM2_ESM.zip › SOURCE DATA 01.0323 E_Verdin/01.0323 O E_Verdin.tif]

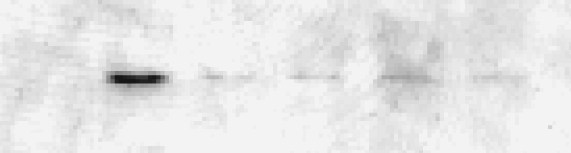

Supplement: Supplementary file 2 — SOURCE DATA 01.0323 E_Verdin [file 44318_2025_612_MOESM2_ESM.zip › SOURCE DATA 01.0323 E_Verdin/01.0323 T E_Verdin.tif]

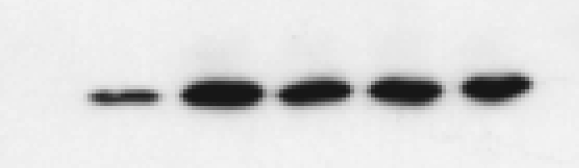

Supplement: Supplementary file 2 — SOURCE DATA 01.0323 E_Verdin [file 44318_2025_612_MOESM2_ESM.zip › SOURCE DATA 01.0323 E_Verdin/01.0323 P E_Verdin.tif]

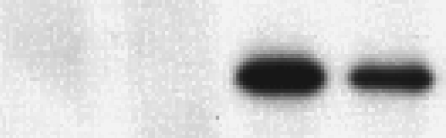

Supplement: Supplementary file 2 — SOURCE DATA 01.0323 E_Verdin [file 44318_2025_612_MOESM2_ESM.zip › SOURCE DATA 01.0323 E_Verdin/01.0323 E E_Verdin.tif]

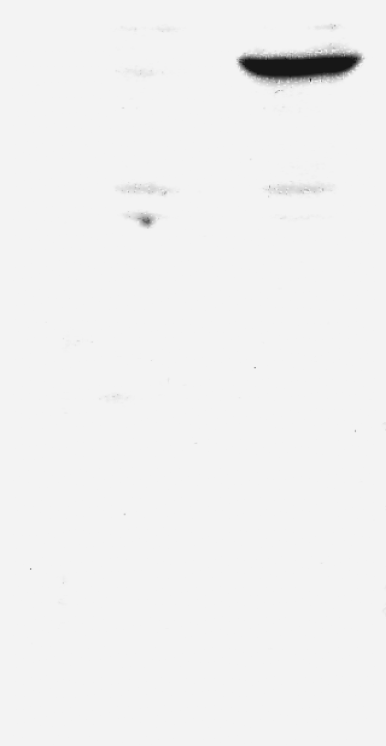

Supplement: Supplementary file 2 — SOURCE DATA 01.0323 E_Verdin [file 44318_2025_612_MOESM2_ESM.zip › SOURCE DATA 01.0323 E_Verdin/01.0323 A E_Verdin.tif]

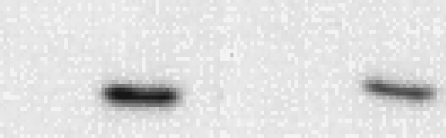

Supplement: Supplementary file 2 — SOURCE DATA 01.0323 E_Verdin [file 44318_2025_612_MOESM2_ESM.zip › SOURCE DATA 01.0323 E_Verdin/01.0323 F E_Verdin.tif]

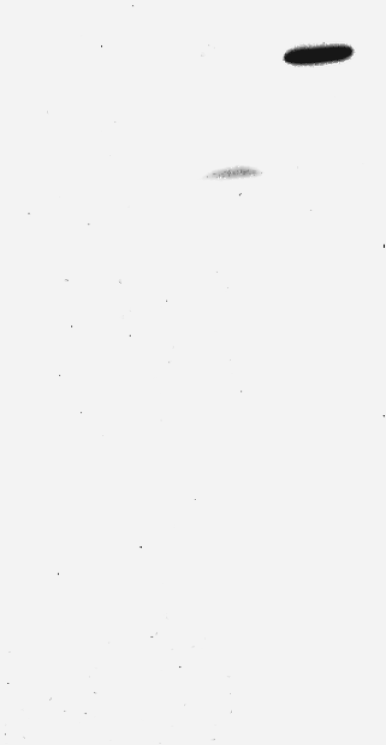

Supplement: Supplementary file 2 — SOURCE DATA 01.0323 E_Verdin [file 44318_2025_612_MOESM2_ESM.zip › SOURCE DATA 01.0323 E_Verdin/01.0323 B E_Verdin.tif]

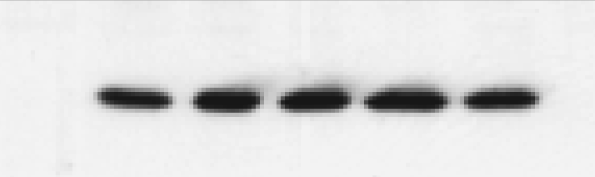

Supplement: Supplementary file 2 — SOURCE DATA 01.0323 E_Verdin [file 44318_2025_612_MOESM2_ESM.zip › SOURCE DATA 01.0323 E_Verdin/01.0323 W E_Verdin.tif]

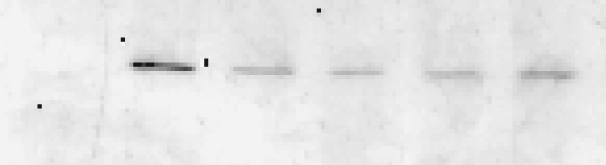

Supplement: Supplementary file 2 — SOURCE DATA 01.0323 E_Verdin [file 44318_2025_612_MOESM2_ESM.zip › SOURCE DATA 01.0323 E_Verdin/01.0323 S E_Verdin.tif]

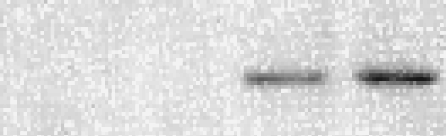

Supplement: Supplementary file 2 — SOURCE DATA 01.0323 E_Verdin [file 44318_2025_612_MOESM2_ESM.zip › SOURCE DATA 01.0323 E_Verdin/01.0323 C E_Verdin.tif]

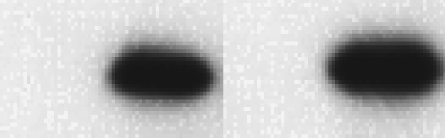

Supplement: Supplementary file 2 — SOURCE DATA 01.0323 E_Verdin [file 44318_2025_612_MOESM2_ESM.zip › SOURCE DATA 01.0323 E_Verdin/01.0323 G E_Verdin.tif]

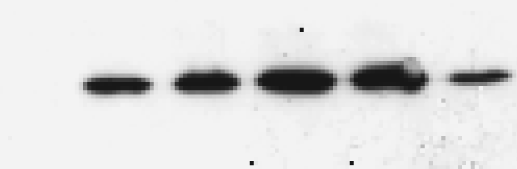

Supplement: Supplementary file 2 — SOURCE DATA 01.0323 E_Verdin [file 44318_2025_612_MOESM2_ESM.zip › SOURCE DATA 01.0323 E_Verdin/01.0323 R E_Verdin.tif]

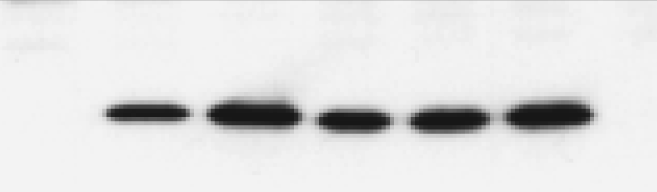

Supplement: Supplementary file 2 — SOURCE DATA 01.0323 E_Verdin [file 44318_2025_612_MOESM2_ESM.zip › SOURCE DATA 01.0323 E_Verdin/01.0323 V E_Verdin.tif]

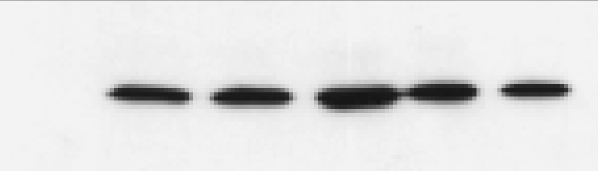

Supplement: Supplementary file 2 — SOURCE DATA 01.0323 E_Verdin [file 44318_2025_612_MOESM2_ESM.zip › SOURCE DATA 01.0323 E_Verdin/01.0323 Q E_Verdin.tif]

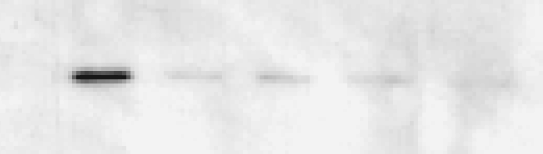

Supplement: Supplementary file 2 — SOURCE DATA 01.0323 E_Verdin [file 44318_2025_612_MOESM2_ESM.zip › SOURCE DATA 01.0323 E_Verdin/01.0323 U E_Verdin.tif]

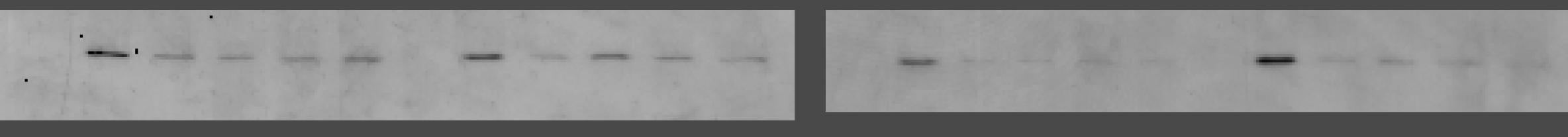

Supplement: Supplementary file 2 — SOURCE DATA 01.0323 E_Verdin [file 44318_2025_612_MOESM2_ESM.zip › SOURCE DATA 01.0323 E_Verdin/01.0323 S,T,U Original.tif]

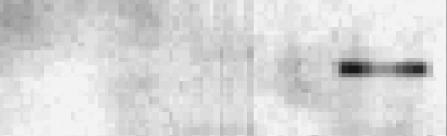

Supplement: Supplementary file 2 — SOURCE DATA 01.0323 E_Verdin [file 44318_2025_612_MOESM2_ESM.zip › SOURCE DATA 01.0323 E_Verdin/01.0323 D E_Verdin.tif]

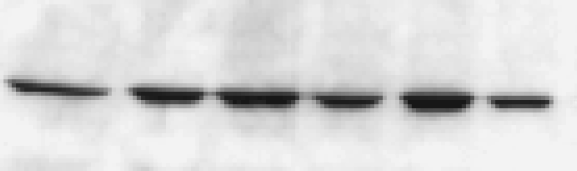

Supplement: Supplementary file 2 — SOURCE DATA 01.0323 E_Verdin [file 44318_2025_612_MOESM2_ESM.zip › SOURCE DATA 01.0323 E_Verdin/01.0323 N E_Verdin.tif]

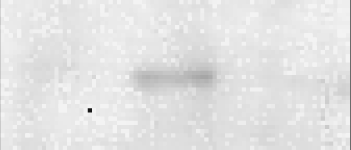

Supplement: Supplementary file 2 — SOURCE DATA 01.0323 E_Verdin [file 44318_2025_612_MOESM2_ESM.zip › SOURCE DATA 01.0323 E_Verdin/01.0323 J E_Verdin.tif]

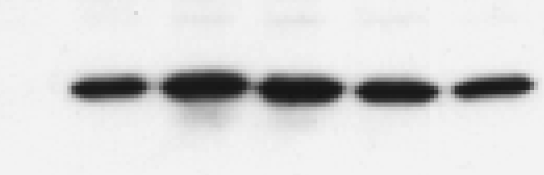

Supplement: Supplementary file 2 — SOURCE DATA 01.0323 E_Verdin [file 44318_2025_612_MOESM2_ESM.zip › SOURCE DATA 01.0323 E_Verdin/01.0323 X E_Verdin.tif]

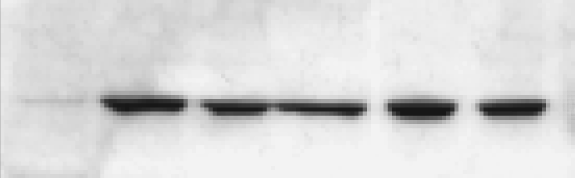

Supplement: Supplementary file 2 — SOURCE DATA 01.0323 E_Verdin [file 44318_2025_612_MOESM2_ESM.zip › SOURCE DATA 01.0323 E_Verdin/01.0323 M E_Verdin.tif]

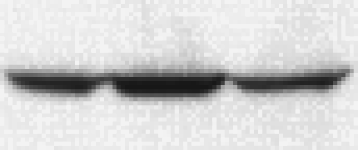

Supplement: Supplementary file 2 — SOURCE DATA 01.0323 E_Verdin [file 44318_2025_612_MOESM2_ESM.zip › SOURCE DATA 01.0323 E_Verdin/01.0323 I E_Verdin.tif]
